# Promising Therapy Candidates for Liver Fibrosis

**DOI:** 10.3389/fphys.2016.00047

**Published:** 2016-02-16

**Authors:** Ping Wang, Yukinori Koyama, Xiao Liu, Jun Xu, Hsiao-Yen Ma, Shuang Liang, In H. Kim, David A. Brenner, Tatiana Kisseleva

**Affiliations:** ^1^Department of Surgery, University of CaliforniaSan Diego, La Jolla, CA, USA; ^2^Department of Medicine, University of CaliforniaSan Diego, La Jolla, CA, USA; ^3^Liver Research Center, Beijing Friendship Hospital, Capital Medical UniversityBeijing, China; ^4^Department of Surgery, Graduate School of Medicine, Kyoto UniversityKyoto, Japan

**Keywords:** liver fibrosis, hepatocytes, cholangiocyte, inflammation, myofibroblasts, early-phase clinical trial

## Abstract

Liver fibrosis is a wound-healing process in response to repeated and chronic injury to hepatocytes and/or cholangiocytes. Ongoing hepatocyte apoptosis or necrosis lead to increase in ROS production and decrease in antioxidant activity, which recruits inflammatory cells from the blood and activate hepatic stellate cells (HSCs) changing to myofibroblasts. Injury to cholangiocytes also recruits inflammatory cells to the liver and activates portal fibroblasts in the portal area, which release molecules to activate and amplify cholangiocytes. No matter what origin of myofibroblasts, either HSCs or portal fibroblasts, they share similar characteristics, including being positive for α-smooth muscle actin and producing extracellular matrix. Based on the extensive pathogenesis knowledge of liver fibrosis, therapeutic strategies have been designed to target each step of this process, including hepatocyte apoptosis, cholangiocyte proliferation, inflammation, and activation of myofibroblasts to deposit extracellular matrix, yet the current therapies are still in early-phase clinical development. There is an urgent need to translate the molecular mechanism of liver fibrosis to effective and potent reagents or therapies in human.

## Introduction

Liver fibrosis is a wound-healing process of the liver in response to repeated and chronic liver injury with distinct etiologies, such as infectious diseases (e.g., viral hepatitis), metabolic derangements (non-alcoholic steatohepatitis), exposure to chemicals (e.g., alcohol liver diseases), or autoimmune diseases (e.g., primary biliary cirrhosis, primary sclerosing cholangitis, and autoimmune hepatitis). The identical morphology characteristics of liver fibrosis are the quantitative and qualitative deposition of extracellular matrix which is produced by myofibroblasts. Myofibroblasts are absent from the healthy liver, accumulate in the injured liver and serve as the principle effector cells of fibrogenesis. Although control and clearance of the underlying causative etiology (e.g., virus eradication or alcohol absence) can slow down fibrosis progression and lead to fibrosis regression, the extensive knowledge on the mechanism leading to liver fibrosis through hepatocyte injury, chaolangiocyte proliferation, inflammation, and activation of myofibroblasts to deposit extracellular matrix has not been translated into effective and potent reagents or therapies in human so far (Hauff et al., 2015). In this review we would like to summarize the current knowledge of targeting each step of the pathologenesis process to relieve liver fibrosis.

## Injury-induced hepatocyte apoptosis

Chronic liver injury, including viral hepatitis and nonalcoholic steatohepatitis (NASH), is typically associated with apoptosis of hepatocytes, which recruit inflammatory cells and promote liver fibrosis. Inactivation of Caspase 1, Caspase 2, or Caspase 3 protects hepatocytes against apoptosis, reduces cytokines involved in inflammatory signaling, and ameliorates fibrogenesis in diet-induced NASH models (Dixon et al., 2012; Machado et al., 2014; Thapaliya et al., 2014). The pan-caspase inhibitor, VX-166 could reduce oxidative stress, inflammation, HSC activation, and extracellular matrix deposition in non-alcoholic steatohepatis mouse (Witek et al., 2009; Anstee et al., 2010). Another pan-caspase inhibitor, IDN-6556, also attenuates hepatic inflammation, hepatic stellate cells (HSCs) activation, and liver fibrosis in murine bile duct ligation (BDL) model (Canbay et al., 2004) and steatohepatitis models (Barreyro et al., 2015). In a clinical study, oral administration of IDN-6556 can reduce aminotransferase activity in chronic hepatitis C patients (Pockros et al., 2007). Now, IDN-6556 has been completed the investigation in randomized, double-blind phase II studies for the treatment of Non-alcoholic fatty liver disease (NAFLD), hepatitis C virus (HCV), and cirrhosis (Table 1). Interestingly, co-administration of an antioxidant (lithospermate B) and caspase inhibitor (nivocasan) can suppress oxidative stress and hepatocyte apoptosis, resulting in a combined effect on reversal of liver fibrosis in rats (Kim do et al., 2013), which provides a better basis for effective therapy to liver fibrosis.

**Table 1:** **Liver injury animal model findings with potential antifibrotic effects**.

**Targets**	**Mechanism**	**Intervention**	**Animal model**
Injured hepatocytes	Pan-caspase inhibitor	VX-166	NAFLD
	Pan-caspase inhibitor	IDN-6556	BDL; NAFLD
Cholangiocyte	Hh signaling antagonist	GDC-0449	Mdr2-/-
Proliferation	Integrin αvβ6 antagonist	Integrin αvβ6 antibody	Ischemia-related biliary fibrosis after orthotopic liver transplantation
	Integrin αvβ6 antagonist	EMD527040	Mdr2-/-
Recruitment of	P2X7 antagonist	A438079	CCl4; acetaminophen
inflammatory cells	CXCR2-FPR1	BOC-1 and DF2156a	acetaminophen
	HMGB1-mediated inflammatory signaling	E5564	ischemia and reperfusion
	TLR9	COV08-0064	acetaminophen
	IL17A	IL17A antibody	S. japonicum; NASH
Nox/ROS signaling	NOX1/NOX4 inhibitor	GKT137831	CCl4; BDL; Fast-food Diet
	antioxidant	Anthocyanin	Acetaminophen; BDL; Alcohol
	antioxidant	β-Lapachone	alcoholic fatty liver disease
Hh signaling	Progenitor response		CCl4; MCD; DDC
Myofibroblasts Activation	TGF-β antibody	1D11	TAA
	CTGF siRNA	NA	CCl4
	Hh signaling antagonist	GDC-0449	BDL; NASH
	Hh signaling antagonist	CYA	BDL
	S1P antagonist	suramin	CCl4; BDL; TAA
	LOXL2 antibody	AB0023	CCl4

*MCD, methionine-choline deficient diet; DDC, 3,5,-diethoxycarbonyl-1,4-dihydrocollidine diet; TAA, thioacetamide*.

## Injury-induced cholangiocyte proliferation

Injury to cholangiocytes recruits inflammatory cells from the blood and activates portal fibroblasts in the portal area, which release molecules to activate and amplify the proliferation biliary progenitor cells (activated cholangiocytes). In BDL cholestatic injury model, bile ductular cells and fibroblastic-appearing cells in the portal area express Hedgehog (Hh) ligands, receptor, and/or target genes (Omenetti et al., 2007). Furthermore, myofibroblasts release soluble Hh ligands that stimulate cholangiocytes to produce Cxcl16 and recruit NKT cells (Omenetti et al., 2009). Hh signaling antagonist GDC-0449 could inhibit liver myofibroblasts and progenitors, relieve liver fibrosis, and even promotes regression of HCC in phospholipid flippase (Mdr2) knockout mice (Philips et al., 2011). Integrin αvβ6, which is another molecular absent in normal liver, promotes proliferation of cholangiocytes and plays functional roles in activating latent TGF-β1. Cholangiocytes exhibit marked increased expression of αvβ6 integrin in thioacetamide (TAA)- and BDL-induced fibrosis, and in human HCV fibrosis and end-stage cirrhosis (Wang et al., 2007; Popov et al., 2008). Inhibition of αvβ6 could attenuate collagen deposition, improve liver function, and retard progression of biliary fibrosis in mouse orthotopic liver transplantation model (Chen et al., 2013). Impressively, a single dose of integrin αvβ6 inhibitor exhibits antifibrogenic and profibrolytic effects (Popov et al., 2008), which might serve as a target for anti-fibrosis therapy.

## Damaged parenchymal cells recruit inflammatory cells to the liver

Endogenous damage-associated molecular patterns (DAMPS) released or leaked from dying hepatocytes act as danger signals recruiting immune cells to the site of injury and initiating inflammatory response (Kubes and Mehal, 2012). A large variety DAMPS have been identified, including nucleic acids, proteins [for example, high mobility group box-1 (HMGB1)], and cellular components [for example, adenosine triphosphate, (ATP)]. It has been shown that ATP released from necrotic hepatocytes generates an inflammatory microenvironment attracting neutrophils to the liver sinusoids via P2X7R, a sensor monitoring the release of ATP at inflammation sites, in a murine hepatic necrosis model by localized thermal injury (McDonald et al., 2010). Neutrophil recruitment is significantly reduced in response to tissue injury either by blocking P2X7 receptor or by genetic deficiency of *P2x7r*^−∕−^ in mice (McDonald et al., 2010). The specific P2X7 antagonist, A438079, could markedly reduce acetaminophen-induced necrosis (Hoque et al., 2012) and alive CCl4-induced liver inflammation and injury (Huang et al., 2014). Although ATP signals initiate neutrophil adhesion to liver sinusoid, it is the chemokines and mitochondrial contain DAMPs, including formyl-peptide receptor 1 (FPR1), that guide and direct neutrophils migration through health tissue toward injured foci (McDonald et al., 2010). CXCR2-FPR1 antagonism can block neutrophil infiltration and hepatotoxicity in acetaminophen-induced liver injury (Marques et al., 2012). These findings suggest blocking P2X7R and/or CXCR2-FPR1 could be a promising therapeutic approach to control liver inflammation.

The HMGB1 is a nonhistone nuclear protein that facilitates regulatory proteins binding to DNA. HGMB1 is constitutively expressed by most of the cells and will be released under injury and death. HGMB1 is highly induced during liver injury (Ge et al., 2014) and contributes to inflammation through binding to TLR4 (McDonald et al., 2015). It is proposed that HMGB1 may not have a direct proinflammatory effect but acts with other proinflammatory mediators such as lipopolysaccharide (LPS; Bianchi, 2009). LPS, a cell-wall component of Gram-negative bacteria, is among the strongest known inducers of inflammation. It has been shown that patients suffered from chronic liver disease had altered gut microbiota and intestinal permeability resulting in releasing bacterial endotoxins, including LPS, to the circulation (Cesaro et al., 2011; Hartmann et al., 2013; Brenner et al., 2015). The leaky gut further induces and stimulates the progression of liver inflammation in addition to the direct injury. As the receptor of HMGB1 and LPS, TLR4 increases TGF-β1 production by Kupffer cells and activates HSCs promoting liver fibrosis (Federico et al., 2015). An inhibitor of TLR4, Eritoran tetrasodium (E5564), can prevent the gut barrier permeability and reduce liver damage by lower IL-6 level and less NF-κB activation in hemorrhagic shock mice with resuscitation (Korff et al., 2013). Pharmacological inhibition of TLR4 by Eritoran tetrasodium ameliorates liver injury through blocking HMGB1-mediated inflammatory signaling in ischemia and reperfusion mice (McDonald et al., 2015) In addition, both bacterial DNA and mitochondrial DNA and nuclear DNA released by damaged hepatocyte can activate TLR9 and induce a number of cellular immune responses (Kubes and Mehal, 2012). A specific antagonist of TLR9, COV08-0064, could limit the sterile inflammation in rat and mouse models of acute liver injury and acute pancratitis (Hoque et al., 2013). So, the antagonists of TLRs provide us new options of combination drug therapy for controlling liver inflammation.

Th17 cells are derived from Naive CD4^+^ T cells, which are rare in normal liver, but accumulate in the portal areas around bile ducts in chronic liver diseases (Wang et al., 2011; Oo et al., 2012). Th17 cells express CCL20, a ligand for CCR6. Using cytokine-treated human cholangiocytes, CCL20 could induce CCR6-dependent migration of Th17 cells (Oo et al., 2012), which might be one of the mechanisms for inflammatory cells recruiting to the liver. IL17 expressed by Th17 cells activates Kupffer cells to produce proinflammatory and profibrotic cytokines for fibrogenesis, and directly increase the expression of collagen, α-SMA, TGF-β in HSCs (Meng et al., 2012). IL-17 monoclonal antibody ameliorates hepatic granulomatous inflammation through downregulation of inflammatory cytokines and recruitment of neutrophils in mouse infected with *Schistosoma japonicum* (*S. japonicum*) larvae (Zhang et al., 2012). And neutralization of IL-17 in NASH mice ameliorates LPS-induced inflammatory cell infiltrating into the liver (Tang et al., 2011). Antibodies against IL17A has been used in phase II-III clinical trials to establish their therapeutic effects in autoimmune diseases (Schuppan and Kim, 2013), yet no information is available for clinical trials on liver fibrosis now.

## Apoptotic hepatocytes activate HSCs through Nox/ROS signaling

Ongoing hepatocyte apoptosis or necrosis leads to increase in ROS production and facilitates HSCs activation and migration, which is one of the characteristics of chronic liver disease that triggers liver fibrogenesis. Nicotinamide adenine dinucleotide phosphate (NADPH) oxidase (NOX) produces reactive oxygen species (ROS) via transferring electron from nicotinamide adenine dinucleotide phosphate to molecular oxygen, which is different from other redox enzymes that produce superoxide as a byproduct. The mammalian NOX family is composed of seven isoforms: NOX1, NOX2, NOX3, NOX4, NOX5, DUOX1, and DUOX2, which are distinctively expressed in specific cell types in the liver. HSCs express three Nox isoforms, Nox1, Nox2, Nox 3 (Paik et al., 2014). HSCs from p47^phox^-deficient mice (without a regulatory component of NOX) fail to generate ROS in response to angiotensin II, platelet derived growth factor (PDGF), leptin, or apoptotic bodies, and p47^phox^-deficient mice demonstrate reduced liver fibrosis after BDL or the hepatotoxin CCl4. (De Minicis and Brenner, 2007; De Minicis et al., 2010). Since NOX1 and NOX4 are expressed in α-SMA positive activated HSCs, GKT137831, a potent dual NOX1/NOX4 inhibitor, attenuates ROS production and inhibits activation of HSCs (Paik et al., 2014). *In vivo* experiments show that GKT137831 could suppress ROS production, liver inflammation and ameliorate CCl4-, BDL-, and Fast-Food Diet-induced liver fibrosis (Aoyama et al., 2012; Jiang et al., 2012; Bettaieb et al., 2015).

Although hepatocyts express many isoforms of Nox, the development of steatosis by a high-fat, methionine and choline-deficient (MCD) diet is independent from Nox activation in hepatocytes. In steatosis, the majority of ROS production may derive from hepatocellular lipid deposition and subsequent peroxidation. Anthocyanin, a plant-derived antioxidant, could reduce oxidative stress, relieve hepatic inflammation, and protect hepatocytes against injury, indicating its potential antifibrotic effects (Choi et al., 2009; Hou et al., 2010; Donepudi et al., 2012). Administration of beta-Lapachone (3,4-dihydro-2,2-dimethyl-2H-naphthol[1,2-b]pyran-5,6-dione), a natural compound extracted from the bark of the lapacho tree (Tabebuia avellanedae), upregulates apoB100 synthesis and lipid mobilization via modulation of NAD(+)/NADH ratio to activate AMPK signaling (Shin et al., 2014). Although vitamin E may reduce the liver oxidative stress and the fibrosis development, administration vitamin E supplementation does not consistently result in protection from liver injury. Multicenter, long-term clinical trials are still needed to evaluate the role of antioxidants in NASH.

## Dying hepatocytes activate hepatic progenitors through Hh signaling

Hh signal released by dying hepatocyte could activate the compensatory outgrowth of hepatic progenitors, which are involved in liver regeneration (Jung et al., 2010). As a Hh target, osteopontin is highly expressed in fibrotic liver tissue and influences the function of hepatic progenitors (Coombes et al., 2015). And neutralization of osteopontin could suppress progenitor cell response and attenuate liver fibrosis in CCl4, methionine-choline deficient diet (MCD) and 3,5,-diethoxycarbonyl-1,4-dihydrocollidine diet (DDC) mice (Coombes et al., 2015).

## Treating liver fibrosis by targeting myofibroblast activation

Chronic inflammation is linked to liver fibrosis through activating the fibrogenetic effector cells, HSCs, portal fibroblasts, bone marrow-derived fibrocytes, and mesenchymal stem cells. HSCs are the major source of hepatic myofibroblasts during development of liver fibrosis under different etiologies (Mederacke et al., 2013). Portal fibroblasts play a less role in the pathogenesis of liver fibrosis than HSCs because they were implicated to pathogenesis of cholestatic liver injury (Wells, 2014). Fibrocytes with dual characteristics of fibroblasts and hematopoietic cells migrate to the injured liver in response to BDL and CCl4-damaged liver and comprise about 5% of the collagen type I expressing myofibroblasts (Kisseleva et al., 2006; Scholten et al., 2011). Bone marrow-derived mesenchymal stem cells could also be recruited to the injured liver and facilitate fibrogenesis (Russo et al., 2006; Li et al., 2009). Although epithelial-mesenchymal transition of hepatocytes and cholangiocytes has been reported to be another origin of myofibroblasts (Omenetti et al., 2007; Zeisberg et al., 2007; Nitta et al., 2008; Syn et al., 2009), recent studies using cell fate mapping detect only minimal or no contribution of EMT by hepatocytes, cholangiocytes, or hepatic progenitors to myofibroblasts (Scholten et al., 2010; Taura et al., 2010; Chu et al., 2011). And, although endothelial cell injury and neovascularization play a critical role in liver fibrosis, the transition of endothelial cells to mesenchymal cells (EndMT) giving rise myofibroblasts is still not definitively resolved. So, anti-fibrotic therapy targeting the myofibroblast activation process of HSCs, portal fibroblasts, fibrocytes, and mesenchymal stem cells might be more useful than blocking EMT or EndMT.

After engulfment of apoptotic bodies, Kupffer cells are stimulated to produce TGF-β1 (Szondy et al., 2003), which is a potent cytokine to activate HSCs, fibrocytes, and mesenchymal stem cells into myofibroblasts (Kisseleva et al., 2006; Li et al., 2009; Meindl-Beinker et al., 2012). Although TGF-β is one of the most potent stimuli of extracellular matrix synthesis, suppressing its expression remains a major challenge of antifibrotic therapy, since systemic blocking of TGF-β1 can provoke inflammation and increase the risk of neoplasia. Neutralization TGF-β in animal models inhibits liver fibrosis and reduces the risk in developing cholangiocarcinoma (Fan et al., 2013; Ling et al., 2013). Fresolimumab (GC1008) is a human anti-TGF-β1 monoclonal antibody that neutralizes all isoform of TGF-β. In patients with advanced malignant melanoma and renal cell carcinoma, fresolimumab demonstrated acceptable safety and preliminary evidence of antitumor activity (Trachtman et al., 2011; Morris et al., 2014; Lacouture et al., 2015). Using radio-labeled (89)Zr-conjugated fresolimumab for PET to analyze TGF-β expression, GC1008 accumulated in primary tumors and metastases in a manner similar to IgG, and (89) Zr-fresolimumab uptake is seen in sites of tumor ulceration and in scar tissue, where TGF-β is highly active (Oude Munnink et al., 2011). Although there is phase II clinical trial ongoing of fresolimumab, optimal strategies are still needed to restrict it to the fibrotic milieu. TGF-β transduces its signal to target genes through the ALK5 ser/thr kinase receptor. GW6604 (2-phenyl-4-(3-pyridin-2-yl-1H-pyrazol-4-yl)-pyridine), an ALK5 inhibitor, inhibits the transcription and deposition of extracellular matrix and improves the deterioration of liver function in mice (de Gouville et al., 2005). Yet, considering of the pleiotropic effects of TGF-β, treatment with an ALK5 inhibitor should be carefully examined to avoid the unwanted effects (de Gouville and Huet, 2006).

As a TGF-β target gene, CTGF is considered as a central mediator which is specific for promoting fibrogenensis. It has been reported that inhibition of CTGF expression by siRNA prevents CCl4-induced liver fibrosis and can induce regression of liver fibrosis (Hao et al., 2014). A human monoclonal antibody to CTGF, FG-3019, has been investigated in various animal models (e.g., liver fibrosis, diabetes, pulmonary fibrosis) and demonstrated a reduction and regression of fibrogenesis. FG-3019 binds to the second domain of human CTGF and is currently under phase II drug investigation for the treatment of liver fibrosis (Lipson et al., 2012; Hauff et al., 2015).

Platelet-derived growth factor (PDGF) is the most potent mitogen for HSC. Imatinib mesylate, a clinically used PDGF receptor tyrpsine kinase inhibitor, attenuates liver fibrosis at early stages, but does not prevent advanced liver fibrosis in animal experiments (Yoshiji et al., 2005; Neef et al., 2006). PTPRO (protein tyrosine phosphatase, receptor type O) shRNA significantly neutralized PDGF-BB-induced HSC proliferation and myofibroblast marker expression through downregulated phosphorylation of extracellular signal-regulated kinase (ERK) and AKT. PTPRO knockout mice [PTPRO(-/-)] have attenuated liver injury, release of inflammatory factors, tissue remodeling, and liver fibrosis in fibrogenesis induced by BDL or carbon tetrachloride (CCl4) administration (Zhang et al., 2015).

Not only does Hh signaling participate in cholangiocyte-chemokine secretion and progenitor response, but also it is involved in liver injury and inflammation after ischemia reperfusion and BDL (Pratap et al., 2010, 2011). Pharmacological inhibition of Hedgehog signaling by vismodegib (GDC-0449) and CYA could attenuate BDL-induced liver fibrosis (Pratap et al., 2012). And vismodegib relieves hepatic inflammation and liver fibrosis in a mouse nutrient excess model of NASH (Hirsova et al., 2013).

Sphingosine 1-phosphate (S1P) is a multifunctional mediator with increased synthesis in the liver following acute and chronic liver injury (Li et al., 2009, 2011), while its concentration remains a relative low level in the bone marrow. This S1P gradient between liver and bone marrow drives the recruitment of bone marrow-derived mesenchymal stem cells into the circulation and into the liver afterwards. Selective S1P receptor antagonist, suramin, has anti-fibrotic effects in CCl4 and BDL liver fibrosis (Li et al., 2009) and hepatoprotective and antitumor activities in TAA-induced liver injury (Tayel et al., 2014).

Inhibitors of receptor tyrosine kinase and Ser/Thr kinase also demonstrate some anti-fibrosis effects. Multitargeted receptor tyrosine kinase inhibitor Sorafenib, which has been approved for the treatment of advanced renal cell carcinoma and hepatocellular carcinoma (HCC), and Sunitinib, can improve experimental hepatic fibrosis, inflammation, and angiogenesis (Tugues et al., 2007; Mejias et al., 2009). SiRNA of transient receptor potential melastatin 7 (TRPM7), a non-selective cation channel with protein serine/threonine kinase activity, attenuates TGF-β1-induced expression of myofibroblast markers, increases the ratio of MMPs/TIMPs, and decrease the phosphorylation of Smad2 and Smad3 associated collagen production (Fang et al., 2013, 2014). Hepatic nuclear factor kappa B (NF-κB)-inducing kinase (NIK), a Ser/Thr kinase, which is increased in injured livers in both mice and humans, induces hepatocyte injury, activates bone marrow-derived macrophages, and leads to liver fibrosis, and this might serve as a candidate for liver fibrosis therapy (Shen et al., 2014).

No matter what the origin of myofibroblasts is, the common feature of them is expressing extracellular matrix. In liver fibrosis, type I collagen is the most prominent increased components of extracellular matrix. The cross-linking of type I collagen is also increased, which is modulated by the matrix enzyme lysyl oxidase-like-2 (LOXL2). Although blocking collagens have unwanted off-target effects, inhibition of LOXL2 by a monoclonal antibody (AB0023) reduces the production of cytokines, attenuates TGF-β signaling, and inhibits the activate fibroblasts (Barry-Hamilton et al., 2010). Similar to AB0023, another humanized monoclonal LOXL2 antibody (GS-6624) is in randomized, double blind, phase II clinical trials to treat NASH and PSC (Schuppan and Kim, 2013).

## Transplantation of mesenchymal stem cells to relieve liver fibrosis

Mesenchymal stem cells, which reside in various tissue, such as bone marrow, umbilical cord blood, adipose tissue and others, can differentiate into multiple cell lineages *in vitro*, including hepatocyte (Ye et al., 2015). Yet, few engraft human cells could be found in the liver after transplantation of human mesenchymal stem cells into the sublethally irradiated NOD/SCID mice exposed to acute or chronic CCl4 injury (di Bonzo et al., 2008). Although endogenous mesenchymal stem cells contribute to liver fibrogenesis during liver injury as described previously, recent studies have demonstrated that exogenous transplantation of mesenchymal stem cells have immunomodulation, inflammation suppression and antifibrogenic effects (Lin et al., 2011; Wang et al., 2014; Christ et al., 2015). Many clinical investigations on the safety and efficacy of mesenchymal stem cells for treatment of liver failure and liver cirrhosis are under phase I and phase II studies (Table 2).

**Table 2:** **Ongoing clinical studies of antifibrotic drugs**.

**Targets**	**Mechanism**	**Intervention**	**NCT no**.	**Disease condition**	**Phase**	**Status**
Injured hepatocytes	Pan-caspase inhibitor	IDN 6556	NCT02230683	Liver cirrhosis with portal hypertension	Phase 2	Complete
	Pan-caspase inhibitor	IDN 6556	NCT02077374	NAFLD	Phase 2	Complete
	Pan-caspase inhibitor	IDN 6556	NCT00088140	Chronic HCV	Phase 2	Complete
	Pan-caspase inhibitor	IDN 6556	NCT02230670	Liver cirrhosis	Phase 2	Active, not recruiting
	Pan-caspase inhibitor	IDN 6556	NCT01912404	Severe AH	Phase 2	Terminated
Cholangiocyte proliferation	hedgehog inhibitor	LDE225	NCT02151864	HCC and liver cirrhosis	Phase 1	Recruiting
Nox/ROS	Anti-oxidant	Vitamin E	NCT01792115	NAFLD	Phase 2	Recruiting
Signaling	Anti-oxidant	Anthocyanin	NCT01940263	NAFLD	Phase 0	Complete
Myofibroblasts	CTGF antibody	FG-3019	NCT01217632	Liver Fibrosis Due to HBV	Phase 2	Active, not recruiting
	LOXL antibody	Simtuzumab	NCT01452308	Liver Fibrosis	Phase 2	Complete
	LOXL antibody	Simtuzumab	NCT01672879	NASH	Phase 2	Active, not recruiting
	LOXL antibody	Simtuzumab	NCT01672853	PSC	Phase 2	Active, not recruiting
Mesenchymal stem cells	Umbilical cord MSCs	Transplantation	NCT01724398	Liver failure	Phase 1	Unknown
					Phase 2	
	Umbilical cord MSCs	Transplantation	NCT01218464	Liver failure	Phase 1	Unknown
					Phase 2	
	Autologous MSCs	Transplantation	NCT01741090	Alcoholic Liver Cirrhosis	Phase 2	Unknown
	Umbilical cord MSCs Bone marrow MSCs	Transplantation	NCT01844063	Liver failure	Phase 1 Phase 2	recruiting
	Autologous MSCs	Transplantation	NCT00420134	Liver failure, cirrhosis	Phase 1	Complete
					Phase 2	
	Autologous MSCs	Transplantation	NCT00956891	Liver failure	Phase 1	Complete
					Phase 2	

## Conclusion

Currently, there are many potential antifibrotic targets obtained from animal experiments (Table 1), yet most of the therapies are still in preclinical evaluation stages (Table 2). Since both animal experiments and clinical studies have revealed that liver fibrosis, even early cirrhosis, is reversible, treating patients by combined therapies on underline etiology and fibrosis simultaneously might expedite the regression of liver fibrosis and promote liver regeneration. In the circumstance that the underline etiology of liver fibrosis could not be eradicated, therapies on liver fibrosis might help restrict the disease progression to cirrhosis and reduce the risk of cirrhosis related complication. In the future, the elucidation of the molecular steps of regression of liver fibrosis might provide new preventive and therapeutic strategies for fibrosis even cirrhosis patients.

## Author contributions

All authors listed, have made substantial, direct and intellectual contribution to the work, and approved it for publication.

### Conflict of interest statement

The authors declare that the research was conducted in the absence of any commercial or financial relationships that could be construed as a potential conflict of interest.
